# The EPINetz Twitter Politicians Dataset 2021. A New Resource for the Study of the German Twittersphere and Its Application for the 2021 Federal Elections

**DOI:** 10.1007/s11615-022-00405-7

**Published:** 2022-06-15

**Authors:** Tim König, Wolf J. Schünemann, Alexander Brand, Julian Freyberg, Michael Gertz

**Affiliations:** 1 Institute of Social Sciences, University of Hildesheim, Hildesheim, Germany; 2 Institute of Computer Science, Heidelberg University, Heidelberg, Germany

**Keywords:** Social Media, Germany, Data, Political communication, Political representatives, Election campaigns, Soziale Medien, Deutschland, Daten, Politische Kommunikation, Politische Repräsentanten, Wahlkampf

## Abstract

This research note introduces the EPINetz Twitter Politicians Dataset, a comprehensive dataset of 2449 Twitter accounts of German parliamentarians, minsters, state secretaries, parties, and ministries on a state, federal, and European Union level for the year 2021. This hand-curated dataset not only provides up-to-date information on elected officials, but it also includes additional variables such as their party affiliation, age, and gender. Furthermore, it provides linkages to additional data sources by providing the accounts’ Wikidata and Abgeordnetenwatch (Parliamentwatch) IDs. While it does not provide actual tweet data, the dataset will be a valuable resource for researchers by providing easy access to elected German politicians. We demonstrate some of the dataset’s uses with an analysis of the 2021 German Federal Elections. The full dataset can be accessed via 10.7802/2415.

**Supplementary Information:**

The online version of this article (10.1007/s11615-022-00405-7) contains supplementary material, which is available to authorized users.

## Abstract (de)

Die Research Note stellt den *EPINetz–Twitter-Politicians-*Datensatz vor, eine umfassende und aktuelle Sammlung der Twitter-Accounts deutscher Parlamentarier:innen, Minister:innen, Staatssekretär:innen, Parteien und Ministerien auf Länder‑, Bundes- und europäischer Ebene für das Jahr 2021. Die insgesamt 2449 händisch kuratierten Accounts sind darüber hinaus mit Variablen wie Parteizugehörigkeit, Alter und Geschlecht annotiert. Zusätzlich enthalten sie die zugehörigen Wikidata- und Abgeordnetenwatch-IDs, um Verbindungen des Datensatzes mit diesen Datenquellen zu vereinfachen. Der Datensatz selbst stellt keine Twitterdaten zur Verfügung, sondern soll es Forschenden ermöglichen, einfach auf hochwertige Daten zu den Twitteraktivitäten politischer Repräsentant:innen in Deutschland zuzugreifen. Wir demonstrieren einige mögliche Anwendungsszenarien für den Datensatz mit einer Analyse der Bundestagswahlen 2021. Der vollständige Datensatz steht unter 10.7802/2415 zur Verfügung.

## Introduction

This research note presents the EPINetz Project’s Twitter Politicians Dataset, a comprehensive overview of institutionalised German politics on Twitter. Over the years, digital media have been recognised as playing an increasingly relevant role in political communication. By now, it has become a widely accepted truth that social media have taken their place among the arbiters of public opinion (Margetts 2019; Jungherr et al. 2020). While assessments of their actual impact on democracy vary (Persily and Tucker 2020), social media have become a cornerstone for politicians’ efforts at campaigning, agenda-setting, and communicating with their constituencies (Bossetta 2018; Dimitrova and Matthes 2018). This can in no small part be attributed to the complex intermedia effects between social and “legacy” media (Jungherr et al. 2019a; Su and Borah 2019; Langer and Gruber 2021). The platform Twitter, in particular, has gained scholarly attention due to its high popularity among politicians and journalists (Dagoula 2019; Molyneux and Mourão 2019), as well as its relatively easy, API–driven (application programming interface) access for researchers (Özkula et al. 2022). Especially during elections, Twitter has become a focal point for both campaigners and researchers (Conway et al. 2015; Jungherr 2016; Zhang et al. 2018). The EPINetz Project’s Twitter Politicians Dataset aims to facilitate political science research on the platform by providing a curated list of politically relevant accounts.

While use of the platform among the general population is relatively sparse in most non–Anglo-Saxon countries, including Germany (Newman et al. 2021), scholars have argued that its popularity among political elites, its relatively low adoption cost, and few topical and formal restrictions to content make it especially well-suited to study political communication. Not only has it been noted that Twitter communications of politicians can be a rich data source comparable to press statements (Sältzer 2022),but it has also been argued that the self-selection of politically interested ordinary users on Twitter comprises an especially valuable subset of the average population when it comes to tracking political debates (Barberá 2015). Furthermore, as with all social media data, it can be argued that its unobtrusive observation of communication yields a higher validity and circumvents certain forms of bias, such as social acceptance or party guidelines, that are present in surveys or (press) interviews (Barberá et al. 2015; Ceron 2017). In this regard, research concerning the generational gap in social media and online news use is proving vital in assessing the potential for generalising these findings (Mangold et al. 2021). In political science, Twitter data have been employed to estimate the ideological positions of ordinary citizens (Barberá et al. 2015; Barberá 2015), between parties (Ecker 2017) and in intraparty politics (Ceron 2017; Sältzer 2022). Furthermore, Twitter data have been utilised as a valid proxy for political issue attention among both citizens and political elites, enabling the study of issue ownership and politicians’ responsiveness to the media and public (Barberá et al. 2019; Ceron et al. 2020; Franzmann et al. 2020). The platform has also been under study for assessing different communication styles and campaign negativity between parties (Evans et al. 2014; Hegelich and Shahrezaye 2015; Russell 2018; Petkevic and Nai 2021). Connected to these questions is research concerned with the fragmentation of political communication into so-called echo chambers and their detrimental effects on the political climate (Barberá et al. 2015; Hegelich and Shahrezaye 2015; Vaccari et al. 2016). Likewise, the assessment of populist rhetoric on Twitter has been under special scrutiny (Ernst et al. 2017; Gründl 2020; Maurer and Diehl 2020), since social media is suspected to empower populist parties (Engesser et al. 2017; Jungherr et al. 2019b). Due to the high volume of political communication, the assumed representativeness of party positions, and their role as a democratic focal point, election campaigns are the focus in the majority of the aforementioned studies (Evans et al. 2014; Hegelich and Shahrezaye 2015; Vaccari et al. 2016; Ceron 2017; Russell 2018; Ceron et al. 2020; Franzmann et al. 2020; Petkevic and Nai 2021). For the German context, a number of studies specifically analyse politicians’ use of the platform during federal election campaigns (Jürgens and Jungherr 2015; Schmidt 2017, 2021; Stier et al. 2018b). In this regard, questions of a changing landscape of political communication arise—both in general terms (Van Aelst et al. 2017; Borucki and Jun 2018) and with a focus on German political institutions (Murphy 2019). During the COVID-19 pandemic, these questions of governmental communication on Twitter have gained new urgency (Rufai and Bunce 2020; Rivas-De-roca et al. 2021).

For all these research approaches, and despite the relatively liberal data access that Twitter grants to researchers, a major obstacle remains. The open architecture, low access barriers, and high anonymity of the platform pose a challenge when attempting to tie observations back to the institutionalised political system. While topic-driven sampling approaches through hashtags and other methods merit credit for producing valuable insights on how discourse on the platform works, critics may claim they have little resemblance to the *actual* political discourse due to, in many countries, the platform‛s low use among the average population (Newman et al. 2021). At the same time, identifying relevant speakers, such as politicians, can prove challenging, as no central databases tracking their social media presences exist. Researchers interested in the communication of political institutions and politicians, then, would have to handpick the relevant Twitter accounts—a task too resource-intensive for many research projects. While some databases exist that compile extensive information on politicians and parliamentarians (Gerring et al. 2019; Göbel and Munzert 2021), they do not provide a comprehensive overview of their social media presence. There are, however, a few databases focussing on politicians’ presence on the Twitter platform in particular. Most notably, the Politicians on Social Media database^1^ holds information on the Twitter accounts of more than 12,000 politicians from more than 154 countries (Haman and Školník 2021). With a more narrow, European focus, the Twitter Parliamentarian Database holds data for 27 countries and 6437 politicians (van Vliet et al. 2020). For the case of Germany, the GESIS institute released comprehensive datasets of candidates running in the 2013, 2017, and 2021 federal elections (Kaczmirek and Mayr 2015; Stier et al. 2018a; Sältzer et al. 2021). However, all these databases only hold information for parliamentarians elected (or running) on a national or federal level. We argue, however, that this only provides a small window into the communications of institutionalised politics on Twitter. For multilevel polities such as Germany, one has to assume that relevant debates are not limited to the federal level but take place on a state level as well. Moreover, depending on the issue at hand, they are also more or less intertwined with debates on the European level. However, a comprehensive database of relevant actors on a state, federal, and European Union (EU) level is still lacking for Germany. Furthermore, as with any database, without proper and constant maintenance, the data provided become outdated sooner rather than later, establishing a constant demand for up-to-date and well-maintained datasets—especially in the fast-moving world of digital communications.

To cater to these needs, we present the EPINetz Twitter Politicians Dataset. As part of the EPINetz Project,^2^ we curate and store information on the Twitter presence of all German parliamentarians on a federal, state, and EU level, as well as German political institutions such as ministries and parties. This up-to-date, hand-curated dataset provides information on a total of 2449 Twitter accounts, along with their office, party affiliation, and, where applicable, additional information such as their term of service, gender, and year of birth. The data provided were collected over the course of 2021, meaning that all parliamentarians and institutions in office during that year were collected. For parliaments reelected during this time, such as the federal parliament in September 2021, we provide data for both the pre-2021 and post-2021 legislative period. While we do not provide actual tweet data, any researcher with access to Twitter data (such as the official API) can use this dataset to retrieve reliable data on German politicians’ Twitter activity during their terms of service. A fine-grained data structure allows for numerous analyses considering differences between the state and federal levels, certain regions, institutions, offices, and parties, as well as personal attributes such as age and gender. Finally, the comprehensive overview of accounts of federal politicians and institutions provides an up-to-date basis for comparative research on a national level. As such, our dataset will prove a valuable resource for research on, but not limited to, election campaigns, fragmentation, populism, ideological positioning, governmental and party communication, issue attention, and intermedia effects on and through Twitter, as well as questions regarding the influence of sociodemographic variables. In the following, we provide a detailed description of the EPINetz Twitter Politicians Dataset, its method of acquisition, and specific variables. Furthermore, we showcase several possible uses by providing a brief analysis of the 2021 German Federal Elections utilising our dataset. Finally, the conclusion provides further applications and avenues for research, as well as highlights the limitations of the data provided. The dataset can be accessed via the GESIS SowiDataNet|datorium (König et al. 2022).^3^

## The EPINetz Twitter Politicians Dataset

For the initial data acquisition, we built on a number of Twitter lists of German politicians in parliament that were curated and made publicly available by Martin Fuchs, aka “Wahlbeobachter” (election observer).^4^ In order to assess this data, we relied on an early, nonpublic version of the Social Media Observatory’s Twitter Parliamentarian Database (Münch et al. 2021).^5^ After the initial evaluation in early 2021, for every state or federal election thereafter, we scanned the official parliament databases for newly elected members and searched for these members’ Twitter accounts by hand. At the same time, we updated their terms of offices as needed, e.g., when voted out of parliament. Note that, due to the initial evaluation taking place in early 2021, office terms are tracked only for those legislative periods stretching into and starting in 2021 (see Online Appendix A for a comprehensive overview). Finally, in late 2021, after federal elections and numerous state elections had been held, we reviewed the data collected by comparing it with the now updated Twitter lists of German politicians in parliament curated by Martin Fuchs. This list of parliamentarians was then supplemented by an in-depth investigation into the Twitter accounts of German ministers, state secretaries, ministries, and parties on both federal and state levels. As to the EU level, given the multinational character of its institutions, we collected data for German Members of the European Parliament (MEPs) only. Manual data collection to retrieve accounts or resolve inconsistencies between data sources followed a standardised procedure: 1) retrieval of information on government or parliament members through official sources; 2) lookup of matching accounts through the Twitter website’s search function; and 3) evaluation of the account’s description, timeline, and displayed name to determine whether it represented the politician or institution in question. Whenever possible, we prioritised politicians’ official over personal accounts. Accounts that were inactive or superseded by another account remained in the database for archival reasons, e.g., when research interest requires retrieving tweets for a longer period of time.^6^ In order to obtain additional sociodemographic variables, we used the Wikidata API.^7^ Specifically, we retrieved information on gender and year of birth for all individual persons, and the *Abgeordnetenwatch* (Parliamentwatch) ID for parliamentarians. When these variables were unavailable on Wikidata, we manually extracted this information from the Abgeordnetenwatch website instead.^8^

The EPINetz Twitter Politicians Dataset comes with a number of variables, allowing for a more fine-grained analysis. Table 1 gives a comprehensive overview. The ID makes sure the same Twitter account associated with different offices (e.g., when a politician is both a member of parliament and a minister), regions (e.g., changing or multiple parliamentary memberships), institutions (e.g., a change from the state to the federal government), or parties (e.g., changes in party membership) can be uniquely identified in its different roles. Due to this representation of multiple roles and contexts, filtering is advised when collecting data to avoid duplicating tweets. When collecting data, it is also strongly advised to utilise the unchanging User_ID rather than a user’s Twitter_handle. The date of reference for a change in offices (the From and Until variables) is the first session of the newly elected parliament after election or the formation of the new government (see Online Appendix A). Note that, while changes in governments (e.g., changes in ministerial positions during legislatures) are represented in our data, we do not comprehensively track changes in parliament or party affiliation in between these dates of reference. The Abgeordnetenwatch_ID represents a person’s unique identifier on the independent, nonpartisan platform Abgeordnetenwatch (Parliamentwatch). The website tracks incumbencies, candidacies, and voting records for all German parliamentarians. It also allows citizens to publicly pose questions to their representatives. The identifier was included to enable further research avenues, e.g., when trying to associate Twitter activity with voting behaviour or responsiveness to citizens’ questions. We also included the matching Wikidata ID (if available) for all accounts. Additional information on the distribution of variables in the data can be found in Online Appendix B.

**Table 1:** Variables in the EPINetz Twitter Politicians Dataset

Variable	Description
ID	A unique identifier, generated over user_id, region, institution, office, and party membership
Official_name	The official name of a person or institution as referred to in official documents or the press. Middle names and titles are dropped
From	Date of a person’s assumption of office. Only goes back to the last legislative period. Not available for parties and ministries
Until	Date of a person’s dismissal of office. Not available for ongoing offices. Never available for parties and ministries
Party	Party affiliation of a person or official party account. Not available for ministries
Region	The political region a person’s, party’s, or ministry’s activities are focused on. One of the German federal states, the federal level (Bund), or the European Union
Institution	The institutional affiliation of an account. State, federal, or European parliament for parliamentarians; state or federal government for minsters, ministries, and state secretaries; “Party” for official party accounts
Office	Political office associated with an account. Refers to their role as parliamentarian, minister, state secretary, or ministry. Accounts associated with parties are split into “Parliamentary Party Group” (for accounts representing parliamentary factions) and “Speaker” (for accounts representing party speakers)
Twitter_name	An account’s name as displayed on Twitter. Subject to change by users
Twitter_handle	An account’s Twitter handle (@handle). Subject to change by users
User_ID	An account’s unique Twitter ID. Cannot be changed by users
Year_of_birth	A person’s year of birth, if available. Never available for parties and ministries
Gender	A person’s self-ascribed gender, if available. Never available for parties and ministries
Abgeordnetenwatch_ID	A person’s ID on the platform Abgeordnetenwatch (Parliamentwatch), if available. Available only for parliamentarians and former parliamentarians
Wikidata_ID	The ID of the associated Wikidata page, if available

## Exemplary Analysis: German Federal Elections 2021

In this section, we present an exemplary study on the Twitter communication of German politicians during the federal election campaign 2021. Therefore, we collected the timelines (all tweets, including retweets, replies to other tweets, and their associated metadata) of all accounts in our dataset for the period between 19 April and 26 September. We thus let our research period start with the day of the nomination of Annalena Baerbock of the Green Party, the second nomination of a lead candidate after the Social Democratic Party (SPD) had already announced that Olaf Scholz was running for Chancellor. The research period “naturally” ends with the closing of ballot boxes at election day, 6 p.m. We further restricted our sample to accounts related to the seven largest parties represented in the 19th German Bundestag and thus to the parties with a realistic outlook to win mandates in the 20th election. These were the Alternative for Germany (AfD; an extreme right-wing party), the Green Party (Bündnis 90/Die Grünen), the Christian Democratic Party (CDU), the Christian Social Union in Bavaria (CSU; the CDU’s Bavarian sister party), the Liberals (FDP), the Left Party (DIE LINKE), and the SPD. Accounts with no party affiliation, i.e., ministries, were dropped for this analysis. To collect the data, we used Twitter’s academic V2 API access, allowing for full archive searching.^9^ Our data contain a total of 426,614 tweets for the observation period, 188,532 (44%) of which are retweets.

### Twitter Usage by Parties During the Election Campaign

**Fig. 1:**
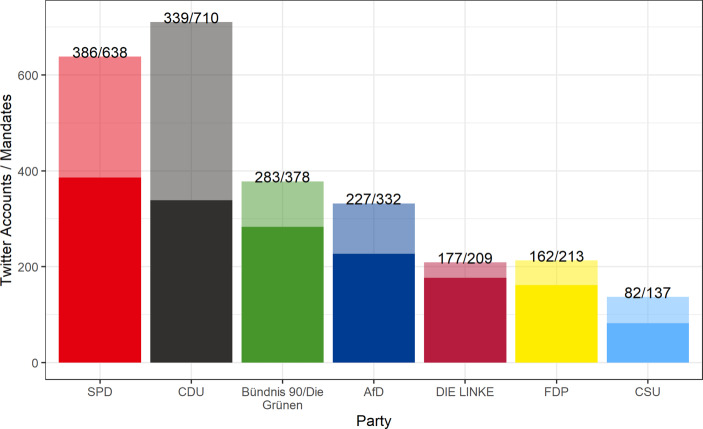
Twitter accounts (total parliamentarians in the dataset) compared to mandates (total of elected parliamentarians across all regions) by party affiliation

Previous research has richly documented the activity differences between parties with respect to certain social media (Jürgens and Jungherr 2015; Schmidt 2017, 2021). These can also be studied using our dataset. First, however, it is important to note that the distribution of Twitter accounts over parties is inherently unbalanced. Figure 1 shows that there are significant differences between a party’s mandates in parliament and its delegates’ Twitter adoption. Politicians and parties vary in the use of Twitter, with lower shares of Twitter account holders and less regular usage among conservative parties. From this perspective, the CDU is especially underrepresented in our dataset. When measured by mandates across all 18 parliaments (regional, federal, EU), the CDU is clearly the largest political party in the country before the 2021 federal elections. However, with less than 50% of its elected politicians on Twitter and their comparatively low activity (see below), the party has relatively low visibility on the platform. This explainable imbalance in the sample should be kept in mind when interpreting the following findings. It shows that Twitter data are not necessarily representative for political discourse in Germany. Nonetheless, Twitter can and should be regarded as an important arena of political online communication.

### Account Features and Variation of Twitter Activity

These imbalances in party representation are even more pronounced when analysing Twitter activity during the campaign period. As shown in Table 2, politicians of Bündnis 90/Die Grünen have been particularly active on Twitter, outnumbering all the other parties by aggregated activity. The other parties on the left of the political spectrum follow in ranks 2 and 3. The CDU, which is second with respect to accounts held, is only fourth with regard to its politicians’ activity. On balance, Twitter seems to be favoured by politicians from the political left, while conservative and right-wing parties are less active on this social media channel. A more differentiated view, also considering the mean activity of party politicians, however, reveals that this comparative insight holds only for the CDU and CSU; the smaller parties FDP and AfD more or less “deliver” the activity that would be expected by their number of accounts—even more so than the larger SPD. Apart from the left–right differentiation, party size and participation in the current government may explain variations in activity, as both the largest German parties, CDU and SPD, were in power during the elections. Besides party preferences in social media activity, generational differences might serve as an explanatory factor for divergent activity. As Table 2 also shows, however, tweet activity for the age groups 35 years and older is considerably higher than for the younger generations. This finding supports recent evidence that, for the German hybrid media environment, the generational gap may be overstated (Mangold et al. 2021). Concerning gender, we see a strong overrepresentation of accounts associated with male politicians in the sample, mirroring gender imbalances in political representation. Female-associated accounts, however, exhibit slightly higher activity than their male counterparts. Unsurprisingly, organisational accounts without sociodemographic features (such as general party accounts) exhibit the highest activity in the sample. With respect to their associated regions, it comes as no surprise that the bulk of tweets comes from accounts associated with the federal level of politics. This is followed by the capital of Berlin, whose state elections were held on the same date as the federal elections. Accounts of politicians on the EU level seem to exhibit a generally higher activity.

**Table 2:** Tweet distribution over variables in the election sample

Variable	Tweets, total	Tweets, %	Accounts	Mean activity	Median activity
*Age group*	*426,614*	*100*	*–*	*–*	*–*
18–24	1282	0.3	8	160.2	86.0
25–34	19,595	4.6	114	171.9	93.5
35–44	117,714	27.6	378	311.4	145.5
45–54	124,523	29.2	476	261.6	123.5
55–64	89,713	21.0	386	232.4	99.0
65+	28,365	6.6	104	272.7	98.0
Organisation	45,422	10.6	83	547.3	377.0
*Gender*	*426,614*	*100*	*–*	*–*	*–*
Male	249,069	58.4	960	259.4	107.0
Female	132,123	31.0	506	261.1	139.0
Organisation	45,422	10.6	83	547.3	377.0
*Party*	*426,614*	*100*	*–*	*–*	*–*
Bündnis 90/Die Grünen	120,612	28.3	317	380.3	205.0
SPD	77,818	18.2	357	218.0	104.0
Die Linke	72,033	16.9	173	416.4	261.0
CDU	51,423	12.1	283	181.7	43.0
AfD	47,853	11.2	182	262.9	76.0
FDP	44,851	10.5	175	256.3	148.0
CSU	12,024	2.8	62	193.9	118.0
*Region*	*426,614*	*100*	*–*	*–*	*–*
Federal	176,204	41.3	495	356.0	181.0
Berlin	52,102	12.2	132	394.7	224.5
EU	34,374	8.1	71	484.1	410.0
Thuringia	23,074	5.4	76	303.6	194.5
North Rhine-Westphalia	21,884	5.1	90	243.2	110.0
Bavaria	18,256	4.3	82	222.6	124.0
Saxony-Anhalt	13,604	3.2	82	165.9	77.5
Schleswig-Holstein	12,850	3.0	35	367.1	80.0
Hamburg	12,570	2.9	76	165.4	83.5
Rhineland-Palatinate	11,057	2.6	61	181.3	53.0
Saxony	10,191	2.4	46	221.5	153.5
Hesse	8544	2.0	77	111.0	58.0
Baden-Württemberg	7247	1.7	62	116.9	32.0
Bremen	6572	1.5	44	149.4	72.5
Brandenburg	6476	1.5	42	154.2	76.0
Lower Saxony	5829	1.4	36	161.9	100.5
Mecklenburg-West Pomerania	4145	1.0	25	165.8	88.0
Saarland	1635	0.4	17	96.2	68.0

### Network Interactions

**Fig. 2:**
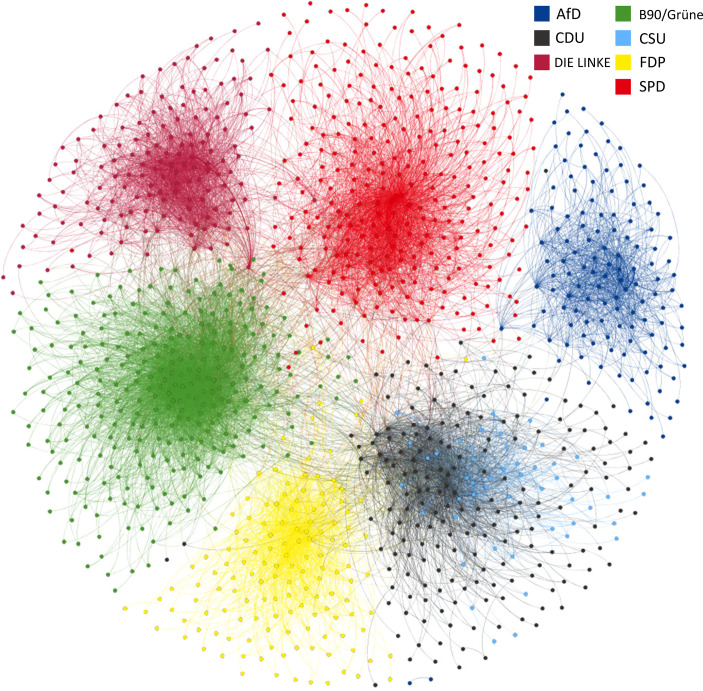
Retweet network. Nodes represent accounts, *coloured* according to party affiliation. *n*(nodes) = 1564; *n*(edges) = 18,846. (Layout: Fruchtermann-Reingold)

Social media like Twitter provide rich opportunities for the study of relational linkages between users. Among other applications, research has made great use of it for empirical inquiry into networks of right-wing extremism (Knüpfer et al. 2020) and for assessing the “transnational arena” in which European Parliament election campaigns take place (Stier et al. 2021). Similar to previous researchers, we stuck to the relational signifiers built into Twitter’s affordance architecture, namely retweets. While there have been divergent views on the meaning of retweets in social communication, in the context of political conflict, retweeting a message can be regarded as a signal of support (Murthy 2012). It can be expected to be strategically motivated in election campaigns insofar as politicians promote the messages of their fellow party members and party organisations while avoiding sharing content of political competitors. For the network analysis of these links, we restricted the nodes to accounts held in the EPINetz Twitter Politicians dataset, dropping all other references and highlighting the comparative relevance of represented politicians. Figure 2 shows the graph for retweeting activity in our sample, with nodes coloured according to party affiliation. As was expected by the assumed strategic use of retweets, party clusters stand out very clearly as separable communities. The Christian Democratic sister parties are an exception in this regard, as their nodes are strongly intertwined. Individual exceptions of nodes appearing separate from their party clusters can be explained by these accounts’ low total number of network interactions, leading to detrimental positioning by the layout algorithm. Most major parties, while identifiable as clusters, are linked by many connections. In contrast, the right-wing populist AfD is singled out and appears at the very margin of the graph.

### References in a Hybrid Media System

**Fig. 3:**
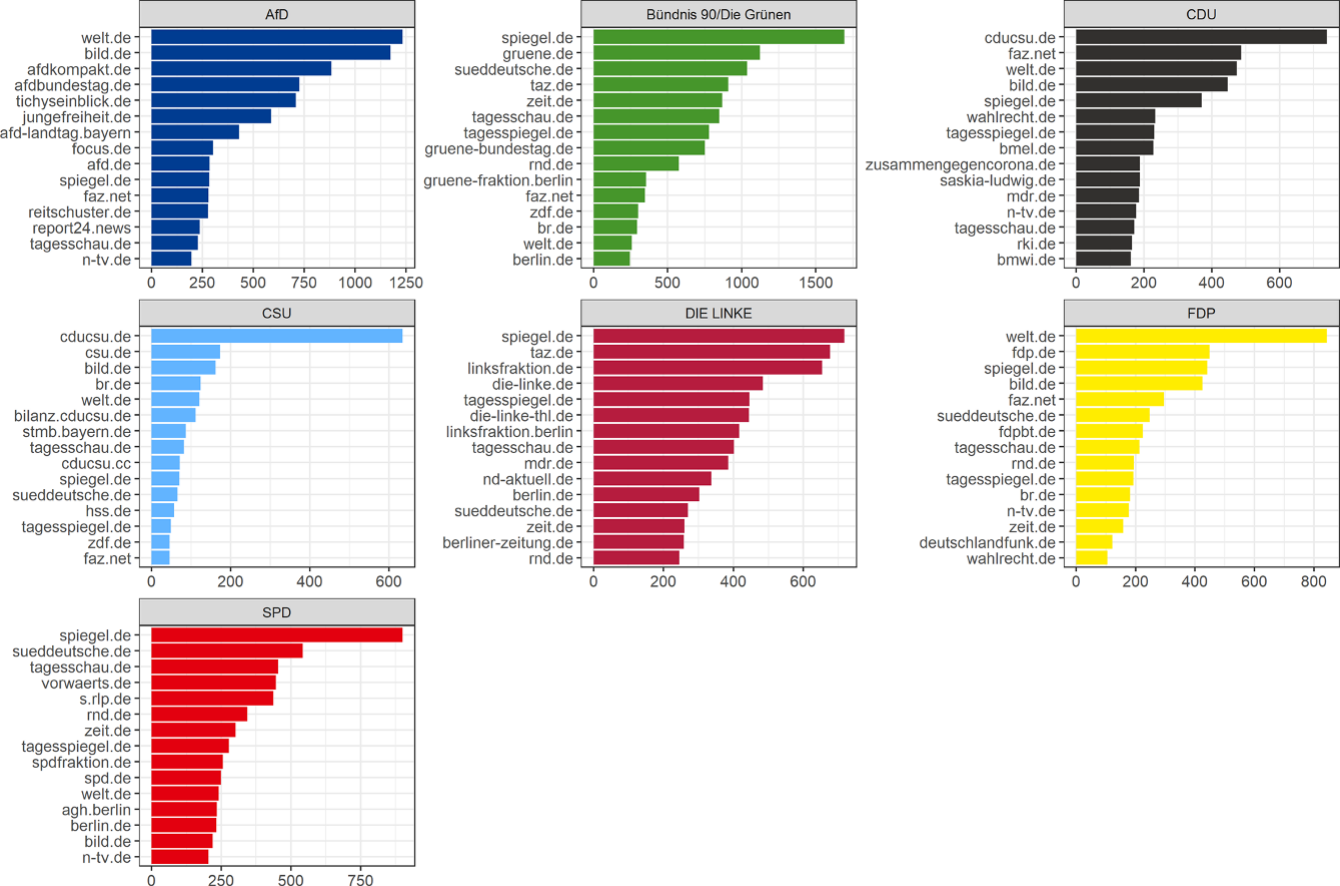
Top 15 most referenced domains by party (*n* = 132,078)

Political communication research has shown great interest in the so-called echo chamber phenomenon. Empirical indicators might be derived from the network analyses presented above, building on theoretical assumptions of homophily regarding party affiliation. Partisan selective media exposure is an alternative conception of the echo chamber (Heft et al. 2021). In our case, party-specific linking patterns can serve as indicators for selective media exposure. Thus, it is of interest which kind of media domains politicians in our dataset refer to. We extracted all URLs shared in the tweets and removed frequent references to Twitter itself—e.g., retweets—or major platforms such as YouTube and Google, totalling 132,078 shared URLs. The remaining URLs were shortened to domains for which we produced the party-wise frequency distributions depicted in Fig. 3. While we find major German news outlets such as the quality press (“FAZ” and “Süddeutsche”) and the public TV news format “Tagesschau” among the top 15 sources for all the parties, there are also some party-specific outlets such as the right-wing news platform “Tichys Einblick” for the AfD and the left-wing newspaper “taz” for the Left and the Green parties.

### Populism in the Election Campaign

**Fig. 4:**
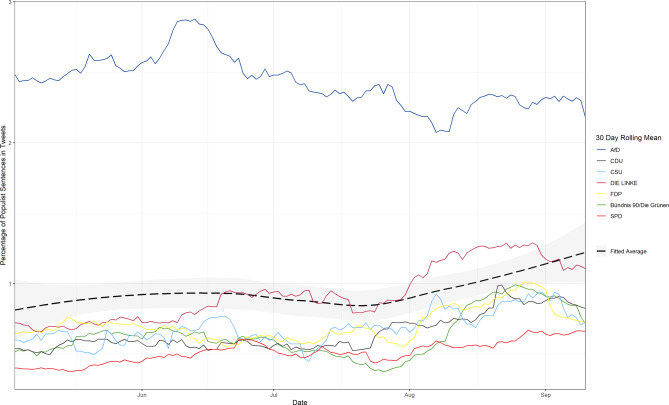
30-day rolling means and fitted average of the percentage of sentences with populist messages. Average fitted via LOESS smoothing

Populist communication on social media has been an increasing concern for political science research, as direct communication via these platforms is suspected to circumvent the power of traditional moderators and gatekeepers, allowing more extremist messages to spread freely (Engesser et al. 2017; Jungherr et al. 2019b). Among similar approaches (e.g., Maurer and Diehl 2020), Gründl (2020) has developed a dictionary-based approach for researching ideational populism for party communication in German-speaking countries. By employing this well-evaluated framework (Gründl 2020), we are able to measure populist communication during the election campaign and compare it between parties. Table 3 gives an overview of the relative amount of populist communication per party. While the percentage of tweets with at least one populist utterance gives a broad overview of the prevalence of populist communication in each party’s election campaign, the percentage of sentences with populist content paints a more fine-grained picture and accounts for different tweet lengths. We can see how the higher share of populist utterances for the AfD confirms its classification as populist, while Die Linke’s slightly above-average values underline its status as a borderline case. Generally speaking, populist messages are not extraordinarily prevalent among politicians on Twitter during the election campaign. These results confirm Gründl’s (2020) findings for the time period of 2014–2020.^10^ For an analysis of populist dynamics in the election campaign, Fig. 4 shows the percentage of sentences with populist messages over time both as 30-day rolling means per party and as a fitted total average. We can see how, interestingly, the AfD spread more populist messages in June, with a downwards trend towards election day. This, and the observable spikes, suggests that short-term issues determine the populist communication of this party more strongly than campaign dynamics. In contrast, for all other parties and the fitted average, we can observe varying increases of populist messages towards election day, reflecting an increasingly heated election battle with uncertain outcomes. This is especially true for the left party Die Linke which, fearing electoral losses, strongly increased its populist messages over the last 6 weeks of the election campaign. These results correspond with findings that propose that campaign negativity may correlate with an increasingly desperate campaign (Petkevic and Nai 2021), suggesting similar patterns for both phenomena.

**Table 3:** Populist messages by parties during the election campaign

Party	Total sentences	Percentage of sentences	Total tweets	Percentage of tweets
AfD	124,638	2.37	47,742	5.96
Die Linke	185,552	0.97	72,286	2.44
FDP	109,996	0.70	45,150	1.66
CSU	31,735	0.69	12,024	1.79
Bündnis 90/Die Grünen	305,361	0.65	120,171	1.60
CDU	128,686	0.64	51,423	1.56
SPD	194,768	0.52	77,818	1.28

## Conclusion

With this research note, we gave an overview of the EPINetz Twitter Politicians Dataset, the variables contained, and the accounts tracked. To illustrate potential use cases for the dataset in political communication research, we presented an analysis of the 2021 German federal elections, providing exploratory insights into the Twitter use of politicians during the election period. We would like to encourage researchers to use the dataset and conduct research beyond the context of election campaigns and the various methods showcased here. Potential use cases are longitudinal studies on politicians’ tweet behaviour, additional uses for the sociodemographic variables provided, and utilisation of the linkages to additional data sources, such as Abgeordnetenwatch for the tracking of voting behaviour in parliament or Wikidata for the collection and analysis of additional data. Current limitations of the dataset are that it does not contain comprehensive tracking of parliamentarians’ mandates during legislative periods and that we provide data only for the legislative periods of 2021. We intend, however, to annually update the EPINetz Twitter Politicians Dataset to continually provide an up-to-date resource for researchers. In this regard, we are looking forward to seeing exciting new applications of the dataset both within and outside the field of political science.

## Supplementary Information

Appendix

## Footnotes

1 https://www.politiciansonsocialmedia.com

2 https://epinetz.de/

3 10.7802/2415

4 http://www.hamburger-wahlbeobachter.de / https://twitter.com/wahl_beobachter

5 Special thanks to Felix-Victor Münch and the team at the Social Media Observatory for granting us access to this work-in-progress database.

6 This produces multiple account entries for politicians with multiple accounts, e.g., for the new Foreign Minister, Annalena Baerbock, whose old Twitter account was superseded and renamed as “@ABaerbockArchiv” when she assumed office.

7 https://www.wikidata.org/. Note that we also use the API to supplement the existing entries with the social media information here collected, allowing researchers to access up-to-date information on German politicians’ Twitter accounts directly via Wikidata.

8 https://www.abgeordnetenwatch.de/

9 See https://developer.twitter.com/en/products/twitter-api/academic-research for details on Twitter’s academic research access. We used the academictwitteR package for the R programming language to access the data (Barrie and Ho 2021).

10 When compared to Gründl’s (2020) results, lower populism in our sample can be explained by the extension of the data to all party politicians, rather than only official party accounts and leaders. Online Appendix C shows the number of populist messages for a subset of accounts mirroring Gründl’s sample. As expected, these numbers show generally higher amounts of populism during the election campaign with its more heated political debates. The CDU’s higher number of populist messages during the election campaign for all subsets can be explained by the Merkel era of relative stability and calm observed by Gründl (2014–2020), being superseded by Laschet’s increasingly desperate (and ultimately failed) 2021 election campaign.
