# Supplementary material for: The EPINetz Twitter Politicians Dataset 2021. A New Resource for the Study of the German Twittersphere and Its Application for the 2021 Federal Elections
Source: Polit Vierteljahresschr. 2022 Jun 15;63(3):529–47. doi: 10.1007/s11615-022-00405-7 (PMC9199468; doi:10.1007/s11615-022-00405-7)
Supplement: Supplementary file 1 — Appendix [file 11615_2022_405_MOESM1_ESM.docx]

Tim König^†^, Wolf J. Schünemann^†^, Alexander Brand^†^, Julian Freyberg^‡^, Michael Gertz^‡^

† University of Hildesheim

‡ University of Heidelberg

The EPINetz Twitter Politicians Dataset 2021

A New Resource for the Study of the German Twittersphere and its Application for the 2021 Federal Elections

# Appendix

## Appendix A: Legislative Periods Covered

## Appendix B: Variable Overview and Distributions

## Appendix C: Populism Measures in the Subsample

## Appendix A: Legislative Periods Covered

Starting dates of legislative periods covered for each region in the dataset. Note that only regions with elections during 2021 have a second period covered. The first session of the newly-elected parliament, not election day, marks the beginning and end of a legislative period for parliaments. For governments, the date of forming the government marks the beginning and end of that period.

**Table 1** Legislative Periods Covered

| **Region** | **Legislative Period I** | | **Legislative Period II (if available)** | |
| --- | --- | --- | --- | --- |
|  | Parliament | Government | Parliament | Government |
| Federal (Bund) | 2017-10-24 | 2018-03-14 | 2021-10-26 | 2021-12-08 |
| European Union | 2019-07-02 |  |  |  |
| Baden-Württemberg | 2016-03-13 | 2016-05-12 | 2021-05-11 | 2021-05-12 |
| Bavaria | 2018-11-05 | 2018-11-12 |  |  |
| Berlin | 2016-10-27 | 2016-12-08 | 2021-11-04 | 2021-12-21 |
| Brandenburg | 2019-09-25 | 2019-11-20 |  |  |
| Bremen | 2019-06-08 | 2019-08-15 |  |  |
| Hamburg | 2020-03-18 | 2020-06-10 |  |  |
| Hesse | 2019-01-18 | 2019-01-18 |  |  |
| Lower Saxony | 2017-11-14 | 2017-11-22 |  |  |
| Mecklenburg-West Pomerania | 2016-10-04 | 2017-07-04 | 2021-10-26 | 2021-11-15 |
| North Rhine-Westphalia | 2017-06-01 | 2017-06-30 |  |  |
| Rhineland-Palatinate | 2016-03-13 | 2016-05-18 | 2021-05-18 | 2021-05-18 |
| Saarland | 2017-04-25 | 2018-03-01 |  |  |
| Saxony | 2019-10-01 | 2019-12-20 |  |  |
| Saxony-Anhalt | 2016-04-12 | 2016-04-25 | 2021-07-06 | 2021-11-16 |
| Schleswig-Holstein | 2017-06-06 | 2017-06-28 |  |  |
| Thuringia | 2019-11-26 | 2020-03-04 |  |  |

## Appendix B: Variable Overview and Distribution

The following tables show the number of accounts in regions and parties (2-3) and the distribution of certain variables over offices, that is, the type of account (4-7). Note how variables such as age and gender are only available for individual persons. While the Abgeordnetenwatch ID is available for all parliamentarians (with the exception of one shared account), it is only available for ministers and state secretaries when they have been elected to parliament at some point in time. NAs in the party variable indicate that this categorisation is not applicable for certain accounts, e.g. ministries.

**Table 2** Accounts per Region

| **Region** | **n** |
| --- | --- |
| Baden-Württemberg | 115 |
| Bavaria | 139 |
| Berlin | 229 |
| Brandenburg | 64 |
| Bremen | 69 |
| EU | 84 |
| Federal | 976 |
| Hamburg | 105 |
| Hesse | 111 |
| Lower Saxony | 69 |
| Mecklenburg-West Pomerania | 71 |
| North Rhine-Westphalia | 150 |
| Rhineland-Palatinate | 115 |
| Saarland | 33 |
| Saxony | 66 |
| Saxony-Anhalt | 119 |
| Schleswig-Holstein | 58 |
| Thuringia | 107 |

**Table 3** Accounts per Party

| **Party** | **n** |
| --- | --- |
| AfD | 278 |
| Allianz Liberaler und Libertärer Europäer | 1 |
| Bayernpartei | 1 |
| Bergpartei | 1 |
| BIW | 1 |
| buendnis21 | 1 |
| Bündnis 90/Die Grünen | 480 |
| Bündnis C | 1 |
| Bürgerbewegung pro NRW | 1 |
| Bürgerrechtsbewegung Solidarität | 1 |
| CDU | 501 |
| Christliche Mitte | 1 |
| CSU | 103 |
| Demokratie in Bewegung | 1 |
| Deutsche Kommunistische Partei DKP | 1 |
| Die Föderalen | 1 |
| Die Grauen | 1 |
| DIE LINKE | 225 |
| DIE PARTEI | 3 |
| Die Republikaner | 1 |
| Die Urbane | 1 |
| dieBasis | 1 |
| Dt. Konservative | 1 |
| Europäische Volkspartei | 4 |
| Familien-Partei Deutschlands | 2 |
| FDP | 257 |
| Freie Wähler | 23 |
| FW | 1 |
| Graue Panther | 1 |
| Klimaliste Baden-Württemberg | 1 |
| Liberal-Konservative Reformer | 2 |
| Liberale Demokraten | 1 |
| LKR | 1 |
| MENSCHLICHE WELT | 1 |
| MLPD | 1 |
| no party affiliation | 7 |
| NPD-Bundesverband | 1 |
| ÖDP | 2 |
| Partei der Humanisten | 1 |
| Partei für Gesundheitsforschung | 1 |
| PdF | 1 |
| Piratenpartei | 2 |
| Renew Europe | 2 |
| SGV-Partei SabinePampel | 1 |
| Sozialistische Gleichheitspartei | 1 |
| SPD | 657 |
| SSW | 5 |
| Team Todenhöfer | 1 |
| Thüringer Heimatpartei | 1 |
| Tierschutzallianz | 1 |
| Tierschutzpartei | 1 |
| UNABHAENGIGE Partei | 1 |
| V-Partei³ | 1 |
| Volt Deutschland | 1 |
| Wir2020 | 1 |
| Zentrumspartei | 1 |
| NA | 88 |

**Table 4** Age Variable by Office, accumulated in Age Groups

| **office** | **age_group** | **n** |
| --- | --- | --- |
| Parliamentary Party Group | NA | 39 |
| Minister | 35-44 | 20 |
| Minister | 45-54 | 61 |
| Minister | 55-64 | 56 |
| Minister | 65+ | 17 |
| Ministry | 55-64 | 1 |
| Ministry | NA | 87 |
| Parliamentarian | 18-24 | 6 |
| Parliamentarian | 25-34 | 194 |
| Parliamentarian | 35-44 | 488 |
| Parliamentarian | 45-54 | 640 |
| Parliamentarian | 55-64 | 607 |
| Parliamentarian | 65+ | 206 |
| Parliamentarian | NA | 1 |
| Speaker | 35-44 | 1 |
| Speaker | NA | 96 |
| State Secretary | 25-34 | 3 |
| State Secretary | 35-44 | 41 |
| State Secretary | 45-54 | 56 |
| State Secretary | 55-64 | 51 |
| State Secretary | 65+ | 8 |
| State Secretary | NA | 1 |

**Table 5** Gender Variable by Office

| **office** | **gender** | **n** |
| --- | --- | --- |
| Parliamentary Party Group | NA | 39 |
| Minister | female | 73 |
| Minister | male | 81 |
| Ministry | NA | 88 |
| Parliamentarian | female | 715 |
| Parliamentarian | male | 1426 |
| Parliamentarian | NA | 1 |
| Speaker | male | 1 |
| Speaker | NA | 96 |
| State Secretary | female | 54 |
| State Secretary | male | 106 |

**Table 6** Availability of the Abegeordnetenwatch ID, by Office

| **office** | **abgeordnetenwatch_id** | **n** |
| --- | --- | --- |
| Parliamentary Party Group | NA | 39 |
| Minister | Available | 137 |
| Minister | NA | 17 |
| Ministry | NA | 88 |
| Parliamentarian | Available | 2141 |
| Parliamentarian | NA | 1 |
| Speaker | NA | 97 |
| State Secretary | Available | 96 |
| State Secretary | NA | 64 |

**Table 7** Availability of the Wikidata ID, by Office

| **office** | **wikidata_id** | **n** |
| --- | --- | --- |
| Parliamentary Party Group | Available | 10 |
| Parliamentary Party Group | NA | 29 |
| Minister | Available | 154 |
| Ministry | Available | 87 |
| Ministry | NA | 1 |
| Parliamentarian | Available | 2140 |
| Parliamentarian | NA | 2 |
| Speaker | Available | 87 |
| Speaker | NA | 10 |
| State Secretary | Available | 160 |

## Appendix C: Populism Measures in the Subsample

Table 8 reports populism measures for the subsample of accounts mirroring Gründl’s (2020) selection of accounts. This subset contains only official federal party accounts and party leaders’ accounts.

**Table 8** Populist Messages during the Election Campaign for Gründl's (2020) Account Sample

| **Party** | **Total Sentences** | **Percentage of Sentences** | **Total Tweets** | **Percentage of Tweets** |
| --- | --- | --- | --- | --- |
| AfD | 4506 | 2.91% | 1442 | 9.02% |
| DIE LINKE | 11124 | 1.35% | 4080 | 3.63% |
| FDP | 7479 | 0.64% | 2636 | 1.82% |
| SPD | 11846 | 0.53% | 4424 | 1.40% |
| CSU | 5703 | 0.53% | 1821 | 1.65% |
| Bündnis 90/Die Grünen | 6054 | 0.43% | 2197 | 1.14% |
| CDU | 3585 | 0.42% | 1293 | 1.16% |
